# Efficacy of electroacupuncture in the treatment of Acute Exacerbation of Chronic Obstructive Pulmonary disease: study protocol for a multicenter, randomized, sham-controlled trial

**DOI:** 10.3389/fmed.2026.1879332

**Published:** 2026-07-13

**Authors:** Shiyu Zhu, Qinmei Yang, Peng Zhang, Jiaxin Xu, Suyun Li, Yang Xie

**Affiliations:** 1 Department of Respiratory Diseases, The First Affiliated Hospital of Henan University of Traditional Chinese Medicine, Zhengzhou, Henan, China; 2 The First Clinical Medical College, Henan University of Traditional Chinese Medicine, Zhengzhou, Henan, China; 3 Henan International Joint Laboratory of Evidence-based Evaluation for Respiratory Diseases, The First Affiliated Hospital of Henan University of Traditional Chinese Medicine, Zhengzhou, China; 4 Collaborative Innovation Center for Chinese Medicine and Respiratory Diseases Co-Construction by Henan Province and Education Ministry of P.R. China, Henan University of Traditional Chinese Medicine, Zhengzhou, China

**Keywords:** acute exacerbation, chronic obstructive pulmonary disease, electroacupuncture, randomized controlled trial, study protocol

## Abstract

**Background:**

Chronic obstructive pulmonary disease (COPD) is one of the most common chronic respiratory diseases worldwide. Recurrent acute exacerbations severely impact patients' quality of life and impose a significant economic burden. The Global Initiative for Chronic Obstructive Lung Disease (GOLD) guidelines recommend acupuncture as an adjunctive therapy for patients with advanced COPD. However, evidence regarding its efficacy during the acute exacerbation phase remains insufficient, particularly from high-quality clinical trials. This study aims to evaluate the efficacy and safety of electroacupuncture in treating patients with acute exacerbation of COPD (AECOPD), establish a standardized electroacupuncture treatment protocol for AECOPD, and preliminarily explore the association between electroacupuncture and airway mucus hypersecretion.

**Methods:**

This is a multicenter, randomized, sham-controlled trial. A total of 336 eligible patients with AECOPD will be recruited and randomly allocated in a 1:1 ratio to either an electroacupuncture group or a sham acupuncture group. Participants will receive treatment once daily for 7 consecutive days and will then be followed up for 13 weeks. The primary outcome measure is the COPD Assessment Test (CAT). Secondary outcomes include the Cough and Sputum Assessment Questionnaire (CASA-Q), the modified Medical Research Council Dyspnea Scale (mMRC), clinical symptom and sign scores, the number of exacerbations, and arterial blood gas. All rating scales will be evaluated at baseline, 4 days after treatment, 7 days after treatment, 5 weeks of follow-up and 13 weeks of follow-up. Arterial blood gas analysis and alveolar lavage fluid collection will be performed at baseline and after 7 days of treatment. The number of acute exacerbations during the follow-up period will be recorded at 5 and 13 weeks of follow-up. Adverse events related to acupuncture will be recorded throughout the study period.

**Conclusion:**

This study will evaluate the efficacy and safety of electroacupuncture in treating patients with AECOPD, establish a standardized electroacupuncture treatment protocol for AECOPD, and preliminarily explore the association between electroacupuncture and airway mucus hypersecretion.

**Clinical trial registration:**

ClinicalTrials.gov [NCT06869525].

## 1 Introduction

Chronic airway diseases represent a major global public health challenge and were ranked among the top four non-communicable diseases by 2023 (1), Chronic obstructive pulmonary disease (COPD) is one of the most common chronic airway diseases and is mainly characterized by recurrent cough, sputum production, and dyspnea (2). According to relevant statistical analyses, COPD is the third leading cause of death globally, with over 3.5 million deaths annually. It is projected that the prevalence of COPD among individuals aged 25 and above will continue to rise, with an increase rate exceeding 20% by 2050 (3). Acute exacerbation of COPD (AECOPD) is characterized by the acute deterioration of respiratory symptoms include dyspnea, cough, and expectoration within 14 days, and it is a key factor in assessing the severity, quality of life, and mortality rate of chronic obstructive pulmonary disease (4). Current management of the acute phase includes pharmacological therapies such as antibiotics, bronchodilators, inhaled corticosteroids (ICS), as well as respiratory support. While these are effective in controlling short-term disease progression, there is no clear evidence regarding their role in preventing early relapse and rehospitalization (5). Furthermore, long-term medication use can result in negative effects such as oral candidiasis, osteoporosis, and diabetes, severely impacting patients' quality of life (6), therefore, further exploration of treatment options is warranted.

As a non-pharmacological therapy, acupuncture has demonstrated significant efficacy in treating respiratory diseases (7, 8). Studies have indicated that acupuncture may improve clinical symptoms, enhance lung function, and reduce rehospitalization rates in patients with COPD. These effects may be associated with immune modulation, suppression of inflammatory responses, and neuroendocrine regulation (9–11). The Global Initiative for Chronic Obstructive Lung Disease (GOLD) guidelines recommend acupuncture as an adjunctive therapy for patients with advanced COPD (2). However, evidence for its efficacy during acute exacerbation is limited and of low quality, partly due to lack of standardization in factors such as needle retention time, acupoint selection, needle type, treatment course, and electroacupuncture frequency in previous studies (12). There remains a lack of large-scale, high-quality clinical research. Therefore, this study aims to evaluate the efficacy and safety of electroacupuncture in treating patients with AECOPD, establish a standardized electroacupuncture treatment protocol for AECOPD, and preliminarily explore the association between electroacupuncture and airway mucus hypersecretion.

## 2 Methods

### 2.1 Trial design and setting

This is a multicenter, randomized, sham-controlled trial. A total of 336 patients with AECOPD will be recruited and centrally randomized in a 1:1 ratio to either an electroacupuncture group or a sham acupuncture group. Participants will receive treatment once daily for seven consecutive days and will then be followed up for 13 weeks. All participants will receive conventional Western medical treatment guided by relevant guidelines. The trial will be conducted across three centers: The First Affiliated Hospital of Henan University of Chinese Medicine, Hebei Provincial Hospital of Traditional Chinese Medicine, and The First Affiliated Hospital of Tianjin University of Traditional Chinese Medicine.

**Figure 1:**
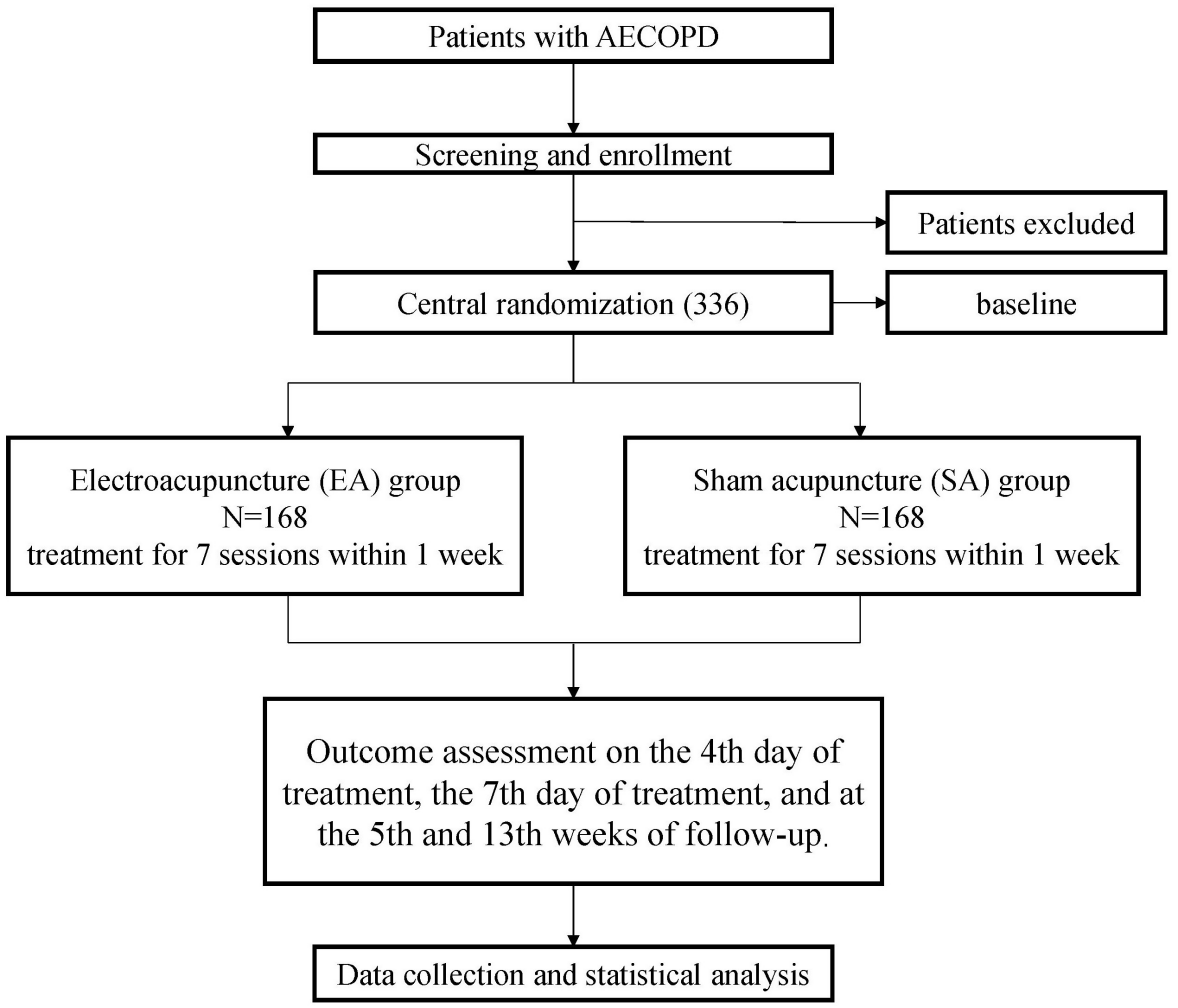
Flow chart of study design.

The study protocol follows the Standard Protocol Items: Recommendations for Interventional Trials (SPIRIT) checklist (Supplementary file 1), also the Standards for Reporting Interventions in Controlled Trials of Acupuncture (STRICTA) checklist (Supplementary file 2) (13). The trial was approved by The First Affiliated Hospital of Henan University of Chinese Medicine Ethics Committee (2024HL-409) and was registered at the ClinicalTrials.gov (NCT06869525). The flowchart of this study design is shown in Figure 1, and the schedule of enrollment, interventions, and assessments is shown in Table 1.

**Table 1:** The schedule of enrollment, interventions, and assessments.

	Enrollment	Allocation	Treatment period							Follow-up	
Timepoint	d-1	d0	d1	d2	d3	d4	d5	d6	d7	Week 5	Week 13
Enrolment:											
Eligibility screen	√										
Informed consent	√										
Baseline assessment	√										
Allocation		√									
Interventions											
EA group			√	√	√	√	√	√	√		
SA group			√	√	√	√	√	√	√		
Assessments:											
CAT	√					√			√	√	√
mMRC	√					√			√	√	√
CASA-Q	√					√			√	√	√
Clinical Symptom and Sign Score	√					√			√	√	√
ABG	√								√		
The number of Acute Exacerbations										√	√
Laboratory test	√								√		
Concomitant medication	√					√			√	√	√
Adverse event			√	√	√	√	√	√	√	√	√

d, day; EA group, electroacupuncture group; SA group, sham acupuncture group; CAT, COPD Assessment Test; mMRC, modified Medical Research Council; CASA-Q, Cough and Sputum Assessment Questionnaire; ABG, Arterial Blood Gas.

### 2.2 Participants and recruitment

Recruitment will be conducted across the three centers through posted advertisements and telephone interviews, primarily within inpatient departments. Potential participants will be assessed and screened by a panel of experts. All eligible participants meeting the inclusion and exclusion criteria will be informed about the study purpose, interventions, treatment duration, benefits, and potential risks. The informed consent form must be signed before randomization. All participant information will be kept confidential.

#### 2.2.1 Inclusion criteria

(1) Patients diagnosed with AECOPD according to the “Global Initiative for Chronic Obstructive Lung Disease (GOLD) 2024 Report (2).”
(2) Aged 40–80 years;
(3) Willing to receive treatment and provide signed informed consent.

#### 2.2.2 Exclusion criteria

(1) Patients with other pulmonary diseases such as lung abscess, pulmonary interstitial fibrosis, or active tuberculosis.
(2) Patients with severe cardiovascular and cerebrovascular diseases (such as malignant arrhythmia, unstable angina, acute myocardial infarction, cardiac function class III or above, stroke, cerebral hemorrhage).
(3) Patients with severe liver diseases and kidney diseases (such as cirrhosis, bleeding due to portal hypertension and esophageal-gastric varices, dialysis, kidney transplantation).
(4) Patients with impaired consciousness, various psychiatric disorders, or others unable to communicate normally.
(5) Pregnant or lactating women.
(6) Individuals who have participated in other clinical trials within 1 month prior to enrollment.
(7) Patients with contraindications to acupuncture (severe allergic and infectious skin diseases, etc.).

#### 2.2.3 Dropout and exclusion criteria

(1) Patients with poor compliance, inability to adhere to treatment, failure to complete the specified course of the trial, or failure to undergo corresponding examinations.
(2) Those who refuse to continue participating in the trial or drop out voluntarily during the trial.
(3) Those who cannot continue the trial due to severe adverse reactions or complications.

### 2.3 Sample size calculation

The primary outcome measure is the COPD Assessment Test (CAT) score. There is currently no established minimal clinically important difference for patients with AECOPD. Therefore, based on preliminary research by our team, the post-treatment CAT score was (14.69 ± 5.23) in the experimental group and (17.08 ± 6.21) in the control group, the expected between-group difference is 2.39. We used the sample size calculation formula for comparing the means of two independent samples to make the estimation: n = $\frac{2{\left(Z_{\alpha }+Z_{\beta }\right)}^{2}*\sigma^{2}}{\delta^{2}}$. Setting a two-sided significance level α = 0.05, statistical power (1–β) = 90%, the calculated sample size per group is approximately 142. Considering a 15% dropout rate, final sample size per group = 142/(1–0.15) ≈ 167.1, therefore, the required sample size per group is approximately 168, totaling 336 participants.

### 2.4 Randomization and allocation concealment

Central randomization will be employed. An independent statistician will use SAS 9.3 software to generate a random sequence stratified by study center, with a block size of 4, ensuring equal allocation to the acupuncture and sham acupuncture groups. Random allocation and concealment will be implemented via a central randomization system operated by a third party (Beijing Depai Software Co., Ltd.). Investigators will obtain a unique, unalterable participant allocation code through this system to ensure allocation fairness.

### 2.5 Blinding

Sham acupuncture will be used as the control condition, and participants will be blinded to group allocation. The acupuncturists administering the interventions cannot be blinded and will be instructed to maintain a neutral demeanor and refrain from any discussion with participants or assessors that might reveal treatment allocation. During treatment sessions, participants will be treated in separate rooms to prevent communication. Outcome assessors and data analysts will be blinded separately. Unblinding will occur after all statistical analyses have been completed. In case of serious adverse events requiring emergency management, unblinding will be performed by an independent Data Monitoring Committee.

### 2.6 Intervention and comparison

**Figure 2:**
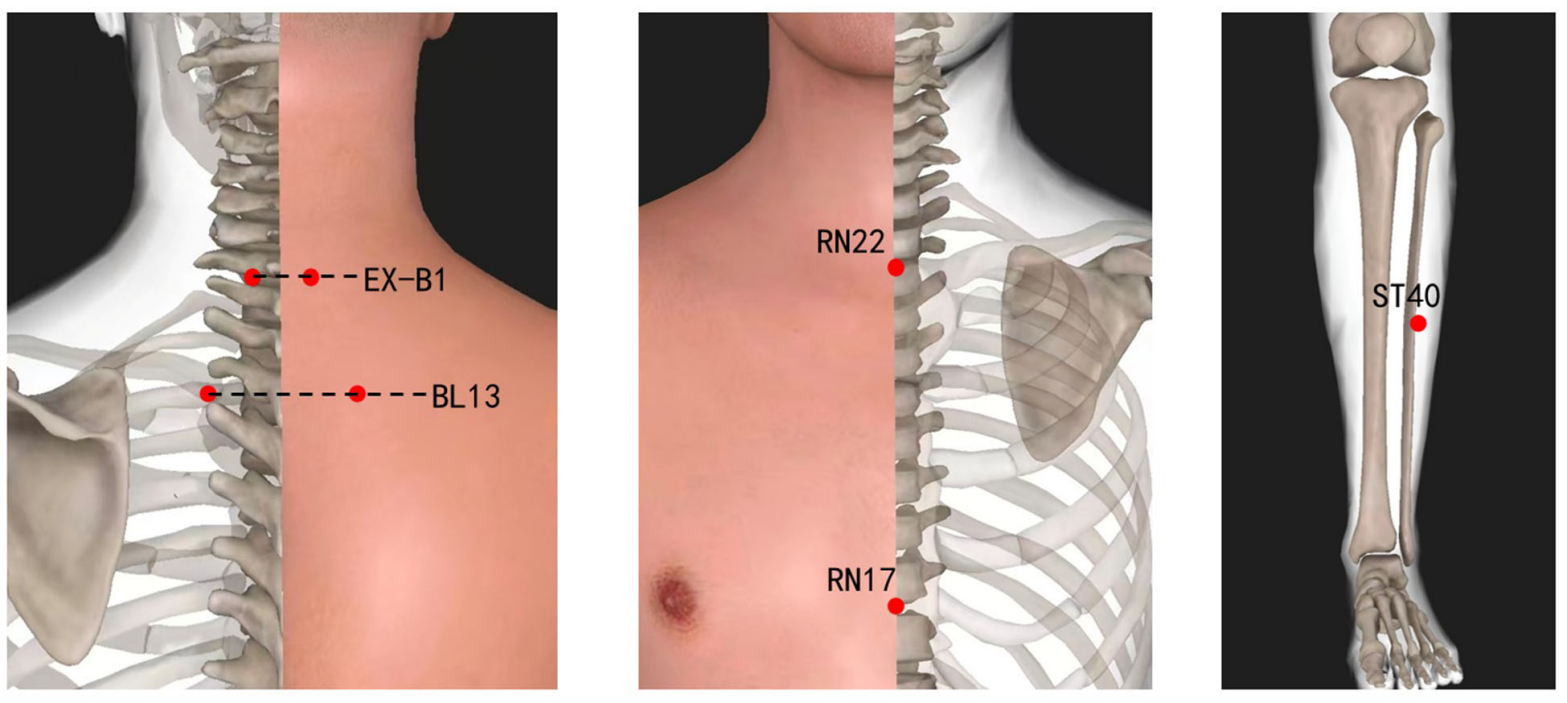
Locations of acupoints. Mannequin images generated from the 3D Body software (v8.8.70), https://www.3dbody.com/.

Participants will be assigned to either the electroacupuncture group or the sham acupuncture group. Acupuncturists will be licensed practitioners with over 5 years of clinical acupuncture experience in tertiary hospitals who will have completed standardized operational training for this study. Disposable acupuncture needles (Huato brand, 0.30 × 40 mm) will be used. The acupoint prescription was determined through literature review and expert consultation, including: Dingchuan (EX-B1), Feishu (BL13), Danzhong (RN17), Tiantu (RN22), and Fenglong (ST40). Dingchuan (EX-B1), Feishu (BL13), and Fenglong (ST40) are selected bilaterally, so, the number of needle insertions is eight. Acupoint localization follows the national standard “Nomenclature and Location of Meridian Points” (GB12346-2021) (14), the locations of acupoints are shown in Table 2 and Figure 2.

**Table 2:** Locations of acupoints.

Acupoints	Locations
Tiantu (RN22)	On the anterior midline, in the center of the suprasternal fossa.
Danzhong (RN17)	On the anterior midline, at the level of the fourth intercostal space, at the midpoint of the two nipples.
Dingchuan (EX-B1)	0.5 cun horizontally next to the depression below the spinous process of the 7th cervical spine.
Feishu (BL13)	1.5 cun horizontally next to the depression below the spinous process of the 3rd thoracic vertebra.
Fenglong (ST40)	Lateral aspect of the lower leg, 8 cun above the tip of the lateral malleolus, lateral to the outer margin of the tibialis anterior muscle.

#### 2.6.1 Electroacupuncture (EA) group

Patients will be in a sitting position with the acupoint areas exposed. After routine skin disinfection, needles will be inserted. The direction and angle of insertion will strictly adhere to standard acupuncture techniques. For Dingchuan and Feishu points, the needle tip will be inserted obliquely slightly inward (toward the spine) to a depth of approximately 0.5–1 cun, avoiding deep insertion that might injure the lung apex. For Tiantu point, the needle will first be inserted perpendicularly 0.2–0.3 cun, then the tip will be directed downward and inserted slowly close to the posterior aspect of the sternum to a depth of approximately 0.5–1 cun. For Danzhong point, the needle will be inserted subcutaneously to a depth of 0.3–0.5 cun. For Fenglong point, the needle will be inserted perpendicularly 1–1.5 cun. Lifting, thrusting, and rotating manipulations will be applied to elicit a distinct sensation of soreness, numbness, distension, or radiation (Deqi). Subsequently, electrodes will be attached for electroacupuncture stimulation using a continuous wave at a frequency of 2 Hz (15), and a current intensity of 1~5 mA, gradually increased to the patient's tolerance level. Treatment duration will be 30 min per session, administered once daily for 7 days, followed by a 13-week follow-up period.

#### 2.6.2 Sham acupuncture (SA) group

Patients will also be in a sitting position with the acupoint areas exposed. Needles will be inserted at locations 5–10 mm lateral to the true acupoints listed above. After skin disinfection, needles will be inserted superficially, just penetrating the skin. Deqi sensation will not be sought. Electrodes will be attached but no current will be delivered. Treatment duration will be 30 min per session, administered once daily for 1 week, followed by a 13-week follow-up period.

#### 2.6.3 Standard Western medical treatment

In addition to acupuncture treatment, patients in both groups will receive standard Western medical treatment with reference to “GOLD (2024)” (2), including medication treatment and respiratory support therapy. The main drugs include three categories: bronchodilators, antibiotics, and glucocorticoids. For bronchodilators, short-acting β2 agonists with or without short-acting anticholinergic drugs via nebulization are selected in the early stage of admission for acute treatment. After the patient's condition stabilizes, long-acting bronchodilators are administered as early as possible for maintenance treatment. Antibiotics will be selected based on laboratory tests or the physician's empirical medication according to local epidemiology, and the recommended course of treatment is 5–7 days. Glucocorticoids will be administered as nebulized budesonide solution or intravenous methylprednisolone 40 mg once daily for 5 days. Specific drugs, dosages, and courses of treatment may be individually adjusted according to the patient's condition. Respiratory support may be given via nasal cannula oxygen inhalation, with a target blood oxygen saturation of 88%-92%, but carbon dioxide retention should be avoided. High-flow nasal cannula oxygen therapy or non-invasive/invasive ventilation will be provided when necessary. All treatment measures and medication details will be recorded in the case report form (CRF), including drug type, dosage, route of administration, frequency, start and stop dates, and treatment duration. For systemic glucocorticoids, the cumulative dose will be converted into a methylprednisolone-equivalent dose where applicable. Respiratory support will also be documented, including conventional oxygen therapy, high-flow nasal cannula oxygen therapy, non-invasive ventilation, invasive ventilation, and duration of use during hospitalization. Any additional co-interventions related to AECOPD management will also be recorded.

### 2.7 Outcome

#### 2.7.1 Primary outcome measure

The primary outcome measure is CAT score. The CAT is a measurement tool commonly used in routine clinical practice to comprehensively assess disease-specific health status and quality of life. It consists of 8 items covering symptoms, activity tolerance, and impact on life, with a score range of 0–40. Higher scores indicate more severe symptoms and greater impacts on health status and daily life (16). CAT will be assessed before treatment, on day 4 of treatment, after treatment completion, and at the 5-week and 13-week follow-up visits.

#### 2.7.2 Secondary outcome measures

(1) Modified Medical Research Council Dyspnea Scale (mMRC): Dyspnea severity will be measured using the mMRC, which consists of 5 grades (0–4). A higher grade indicates more severe dyspnea (17). mMRC will be assessed before treatment, on day 4 of treatment, after treatment completion, and at the 5-week and 13-week follow-up visits.
(2) Cough and Sputum Assessment Questionnaire (CASA-Q): The CASA-Q comprises 4 domains and includes 20 items. Each item is scored from 0 to 4, with reverse scoring based on symptom severity. For each domain, scores are calculated using the following algorithm: (sum of item scores) / (sum of item score ranges)^*^100, resulting in domain scores ranging from 0 to 100. Higher scores indicate milder symptoms and less impact (18). CASA-Q will be assessed before treatment, on day 4 of treatment, after treatment completion, and at the 5-week and 13-week follow-up visits.
(3) Clinical Symptom and Sign Score: This includes seven items: cough, sputum production, wheezing, chest tightness, shortness of breath, fatigue, and cyanosis. Each item is scored from 0 to 3, where 0 represents no symptoms. Higher scores indicate more severe symptoms. This score will be assessed before treatment, on day 4 of treatment, after treatment completion, and at the 5-week and 13-week follow-up visits.
(4) The number of Acute Exacerbations: AECOPD is defined as the progressive deterioration of a patient's symptoms over a period of several days, which typically requires outpatient consultation or hospitalization, accompanied by pharmacological intervention and oxygen therapy. The number of acute exacerbations during the follow-up period will be recorded at weeks 5 and 13.
(5) Arterial Blood Gas (ABG): ABG is an important index for assessing the severity of exacerbations. Arterial blood (1–2 ml) will be drawn from the radial or brachial artery without supplemental oxygen (FiO_2_ set at 20.9%). Parameters measured will include pH, partial pressure of oxygen (PaO_2_), partial pressure of carbon dioxide (PaCO_2_), and the oxygenation index. ABG will be performed before and after treatment.
(6) Laboratory Indicators: Collection of bronchoalveolar lavage fluid will be performed only in patients enrolled at the First Affiliated Hospital of Henan University of Chinese Medicine. Not all randomized patients receive this procedure, bronchoalveolar lavage fluid can be collected after patients undergo safety assessment by respiratory specialists and given written informed consent. This procedure can't be conducted on patients with severe hypoxemia, hemodynamic instability, high bleeding risk or other contraindications for bronchoscopy. ELISA kits will be used to measure levels of mucins and inflammatory factors in the BALF, including MUC5B, MUC5AC, and IL-13. BALF will be collected once before and once after treatment.

### 2.8 Safety assessment and adverse events

Safety indicators include complete blood count, urinalysis, electrocardiogram, liver function, and kidney function tests. These will be monitored and recorded once before and once after treatment. The management of adverse events during treatment will follow the national standard “Management of Adverse Events in Acupuncture” (GB/T 33415-2016). For minor events like local bleeding or mild pain, acupuncturists will apply pressure to stop bleeding and observe the painful area. For severe adverse events such as needle breakage or fainting, patients will be promptly transferred to the emergency department. Emergency department staff will provide symptomatic treatment based on the specific situation, closely monitor the patient's condition, and conduct follow-up examinations to ensure recovery.

For patients receiving bronchoalveolar lavage fluid collection, the feasibility and safety of bronchoscopy and bronchoalveolar lavage fluid collection must be assessed prior to each procedure. Vital signs and blood oxygen saturation shall be continuously monitored during and after the procedure. If procedure-related adverse events occur, including transient hypoxemia, hemorrhage, bronchospasm, fever or other discomforts, timely management shall be implemented in accordance with the hospital's safety specifications for bronchoscopic procedures. Subjects reserve the right to decline or terminate bronchoalveolar lavage fluid sampling at any time, and such decision will neither affect their continued participation in this study nor their access to routine standard medical treatment.

Any adverse event, regardless of its relation to the intervention, must be reported promptly and recorded in the Case Report Form (CRF). Serious cases must be reported to the Project Management Office and the Ethics Committee of The First Affiliated Hospital of Henan University of Chinese Medicine within 24 h, and participation in the trial will be terminated. In the event of an adverse event, participants will immediately receive appropriate treatment and may decide to withdraw from the trial. Participants who withdraw will continue to be investigated and followed up, and their outcomes will be recorded accordingly.

### 2.9 Trial quality control

This research protocol has undergone multiple rounds of review and revision by experts in the fields of acupuncture, respiration, and statistics. This study will be conducted in multiple centers, in order to ensure the consistency of the implementation of the plan across all centers, before the start of the research, all investigators and staff will receive unified standardized training and passe the assessment, including training on the research process, operational specifications, and data entry. Also, standardized training for acupuncturists on the location of acupoints and non-acupoints, as well as the operation of acupuncture and sham acupuncture. An independent quality control team will be established to conduct regular monitoring and irregular spot checks, and meetings will be held promptly to discuss and resolve any problems identified.

### 2.10 Data management and monitoring

During the trial, a clinical trial electronic data capture system will be used for data entry and data management, with strict implementation of randomization and allocation concealment. All participants' data will be reserved in the EDC system, and only data monitors have access to this data. Researchers will not be allowed to modify or access the data until data collection is completed. After data collection is finished, independent statisticians will perform data cleaning and verification, remove invalid data, and ensure the authenticity, completeness, and reliability of the data.

### 2.11 Statistical analysis

Data analysis will be performed by an independent statistician using SPSS 26.0 statistical software (IBM SPSS Statistics, IBM Corp., Armonk, NY, USA). All tests will be two-sided, with a significance level set at α = 0.05. Statistical analysis will follow the Intention-To-Treat (ITT) analysis, with the PP set for sensitivity analysis. All randomized participants will be included in the analysis set. Continuous variables conforming to a normal distribution will be described as mean ± standard deviation; otherwise, median and interquartile range will be used. Categorical variables will be described using frequencies and percentages. Baseline differences between groups will be compared using *t*-tests or Mann–Whitney *U* tests for continuous variables, and chi-square tests or Fisher's exact tests for categorical variables. Repeated measures analysis of outcome scale scores will be performed using mixed-effects models, with group, time, and their interaction as fixed effects, participant as a random intercept, and baseline values as covariates. The number of acute exacerbations will be analyzed using a negative binomial generalized linear mixed-effects model or Poisson regression model. Arterial blood gas results will be compared between groups after treatment using analysis of covariance (ANCOVA), adjusted for baseline values.

All concomitant medications and respiratory support measures will be recorded at the individual-patient level and summarized by different group to assess potential between-group imbalance. If clinically relevant between-group imbalances are observed in key co-interventions, such as systemic glucocorticoid exposure, bronchodilator, or respiratory support, adjusted sensitivity analyses will be performed by including these variables as covariates in the corresponding statistical models.

Missing data will be documented with amount, timing, and reasons. The primary analysis will use multiple imputation under the missing-at-random assumption, including treatment group, baseline outcome values, available follow-up values, disease severity, adverse events, and major co-interventions in the imputation model. Complete-case analysis will be performed as a sensitivity analysis. In addition, pattern-mixture models and tipping-point analysis for the primary outcome will be conducted to assess the robustness of the findings under potential missing-not-at-random assumptions.

## 3 Discussion

COPD is a major global disease, and its acute exacerbations are associated with critical illness (19). Annually, 22%−40% of COPD patients experience at least one acute exacerbation, and 9%−16% experience multiple exacerbations (20), The combined readmission rates within 30 and 90 days are 11% and 17% (21), Recurrent acute exacerbations impose a significant economic burden and high mortality risk in patients with COPD (22), Therefore, during the acute exacerbation phase, the key therapeutic goals are to control disease progression, improve symptom severity, and reduce the risk of recurrent exacerbations. While some studies have confirmed the efficacy of acupuncture for COPD (23, 24), there remains a lack of high-quality clinical evidence and standardized technical protocols.

Clinically, COPD primarily manifests as cough, sputum production, and dyspnea, which are closely related to excessive mucus secretion and accumulation in the airways. MUC5AC and MUC5B are the two major secretory airway mucins constituting the airway mucus layer (25). Related studies stimulating airways with IL-13 found that approximately three-quarters of cells co-expressed MUC5AC and MUC5B (26), indicating a close relationship between the production of airway inflammatory factors and the state of mucus hypersecretion. Numerous experimental studies have demonstrated that acupuncture can reduce airway inflammation, but research on its effect on mucus secretion is limited. Related animal experiments suggest that acupuncture can inhibit mucus secretion by airway epithelial cells, thereby alleviating airway inflammation and lumen obstruction in COPD rats (27). Therefore, this study aims to evaluate the efficacy and safety of electroacupuncture in treating patients with AECOPD, establish a standardized acupuncture protocol for AECOPD, and explore the association between acupuncture and airway mucus hypersecretion.

The trial will be conducted in inpatient departments, and will enroll hospitalized patients. Acute exacerbations of COPD are typically controlled within 7–14 days. To ensure the integrity of the intervention course, the treatment period is set at 7 days. Assessment on treatment day 4 will mainly involve symptom rating scales to evaluate the early symptomatic effect of electroacupuncture during the acute exacerbation phase. The CAT score positively correlates with disease progression and severity in COPD patients and comprehensively covers main symptoms such as cough, sputum, wheezing severity, and quality of life (28). Therefore, we have adopted the CAT score as the primary outcome measure to evaluate the clinical efficacy of electroacupuncture in patients with AECOPD. The typical clinical manifestations of airway mucus hypersecretion are cough and sputum production. Accordingly, we use the CASA-Q questionnaire for assessment. The CASA-Q covers four domains: cough symptoms, cough impact, sputum symptoms, and sputum impact. Compared to other questionnaires, it not only measures the occurrence of cough and sputum symptoms but also reflects their impact on daily life, making it more targeted and comprehensive (18). Simultaneously, we employ ABG to assess the degree of hypoxia and carbon dioxide retention in COPD patients. Furthermore, the frequency of recurrent acute exacerbations will be recorded to judge disease prognosis following acupuncture treatment. Additionally, adverse reactions will be documented in detail to demonstrate the safety of acupuncture.

The primary pathological features of COPD are airway inflammation and mucus hypersecretion. Excessive production of pro-inflammatory cytokines and recurrent airway inflammation can lead to airway mucosal damage and remodeling (29). Stimulated by factors such as infection and smoke, airway epithelial cells and macrophages produce inflammatory factors, inducing secondary goblet cell hyperplasia and increased mucus synthesis and secretion (30), In addition, airway inflammation impairs mucociliary clearance and alters the biophysical properties of mucus, further exacerbating airway infection and creating a vicious cycle (31). The effect of these processes may lead to mucus plug formation, further causing airway narrowing, airflow limitation, worsening dyspnea, and in severe cases, respiratory failure (32). Studies have shown that COPD patients with airway mucus hypersecretion experience a faster decline in lung function, have a 2–4 times higher risk of acute exacerbations and hospitalization, and face a 2.5–11 times increased risk of mortality (33).

The acupoint prescription for this study was determined through literature review and expert consultation, with priority given to points frequently recommended in the expert questionnaires. The selected points for AECOPD treatment are Dingchuan (EX-B1), Feishu (BL13), Fenglong (ST40), Danzhong (RN17), and Tiantu (RN22). Danzhong (RN17), the influential point for Qi, is located on the chest where ancestral Qi gathers. It can soothe the chest, regulate Qi, and harmonize the flow of Qi throughout the body (34). Feishu (BL13), the Back-Shu point of the lung, can disperse lung Qi, relieve wheezing, and tonify lung Qi. It is one of the most commonly used points for treating respiratory system diseases (35), Anatomically, Feishu corresponds to the surface projection of the lungs (36), Previous research has found that acupuncture at Feishu can inhibit the synthesis and release of acetylcholine to alleviate airway inflammation (37). Dingchuan (EX-B1) is an empirical point for relieving wheezing, with the function of regulating lung Qi, stopping cough, and calming wheezing. Acupuncture at Dingchuan (EX-B1)can reduce serum levels of inflammatory cells and airway hyperresponsiveness (38). Tiantu (RN22), located on the Conception Vessel (Ren Mai), connects the throat above and the lung system below, showing significant anti-wheezing effects and effectively treating various lung diseases (39). Phlegm is a primary pathological factor in COPD, present throughout the disease course. Fenglong (ST40), the Luo-Connecting point of the Stomach Meridian of Foot-Yangming, is traditionally regarded as a key point for resolving phlegm. This study employed a non-meridian, non-acupoint, shallow insertion method for the sham acupuncture control group. Currently, in clinical acupuncture research, there is no consensus on how to design sham acupuncture controls, with two main approaches: invasive and non-invasive (40). Commonly used non-invasive sham needles such as Park needles and Streitberger needles, which contain internal mechanisms that automatically retract upon skin contact, providing participants with a certain degree of tactile sensation. The external sheath serves as a visual barrier, thereby ensuring participant blinding. However, with the increasing application of traditional Chinese medicine, more patients are undergoing acupuncture treatment, making it difficult to maintain blinding due to differences in sensory experience between real and simulated acupuncture (41). Moreover, studies have shown that even non-invasive methods may still generate tactile stimulation, activate the somatosensory system, and produce therapeutic effects (42). Additionally, these sham devices require adhesion to acupoints for fixation, making them unsuitable for areas such as the face, head, extremities, or regions with uneven skin surfaces. The Tiantu (RN22) point, located on the suprasternal fossa, presents particular operational challenges. These devices also make it difficult to perform needling at a specific angle, however, Danzhong (RN17), Feishu (BL13), and Dingchuan (EX-B1) require oblique insertion in this study.

## 4 Limitations

First, this study uses penetrating sham acupuncture as the control group and does not include a blank control (no treatment) group. A systematic survey indicates that penetrating sham acupuncture possesses certain physiological effects, which may lead to smaller effect size estimates (43). Although non-acupoint locations, superficial insertion, avoidance of Deqi sensation, and absence of electrical stimulation are used to minimize specific acupuncture effects, the sham acupuncture intervention cannot be regarded as completely physiologically inert. Therefore, the sham acupuncture group in this trial should be interpreted as a minimally active control rather than a pure placebo control. Future studies may consider including an additional group receiving standard Western medical treatment alone to better distinguish the specific effects of electroacupuncture from non-specific needling and contextual effects. Second, in this study, the participants, outcome evaluators and statisticians will be blinded. However, due to the nature of acupuncture, it is impossible to blind the acupuncturists. Therefore, the potential for performance bias impacting the results cannot be excluded.

## 5 Conclusion

This trial will provide evidence regarding the efficacy of acupuncture in treating Acute Exacerbation of Chronic Obstructive Pulmonary disease and preliminarily explore the association between acupuncture effects and airway mucus hypersecretion, offering a reference for clinical practice.
